# The essentials of developmental apoptosis

**DOI:** 10.12688/f1000research.21571.1

**Published:** 2020-02-26

**Authors:** Anne K. Voss, Andreas Strasser

**Affiliations:** 1 Walter and Eliza Hall Institute of Medical Research, Parkville, Victoria, Australia; 2 Department of Medical Biology, The University of Melbourne, Melbourne, Victoria, Australia

**Keywords:** Embryo, fetus, development, programmed cell death, apoptosis, BIM, PUMA, BID, BMF, NOXA, BIK, BAD, HRK, BCL-2, MCL-1, BCL-XL, BCL-W, A1, BAX, BAK, BOK, APAF-1, caspases

## Abstract

Apoptotic cells are commonly observed in a broad range of tissues during mammalian embryonic and fetal development. Specific requirements and functions of programmed cell death were inferred from early observations. These inferences did not hold up to functional proof for a requirement of apoptosis for normal tissue development in all cases. In this review, we summarize how the appraisal of the importance of developmental apoptosis has changed over the years, in particular with detailed functional assessment, such as by using gene-targeted mice lacking essential initiators or mediators of apoptosis. In recent years, the essentials of developmental apoptosis have emerged. We hypothesize that apoptosis is predominantly required to balance cell proliferation. The two interdependent processes—cell proliferation and apoptosis—together more powerfully regulate tissue growth than does each process alone. We proposed that this ensures that tissues and cell populations attain the appropriate size that allows fusion in the body midline and retain the size of cavities once formed. In addition, a limited number of tissues, albeit not all previously proposed, rely on apoptosis for remodeling, chiefly aortic arch remodeling, elimination of supernumerary neurons, removal of vaginal septa, and removal of interdigital webs in the formation of hands and feet.

## Introduction

**Figure 1.**
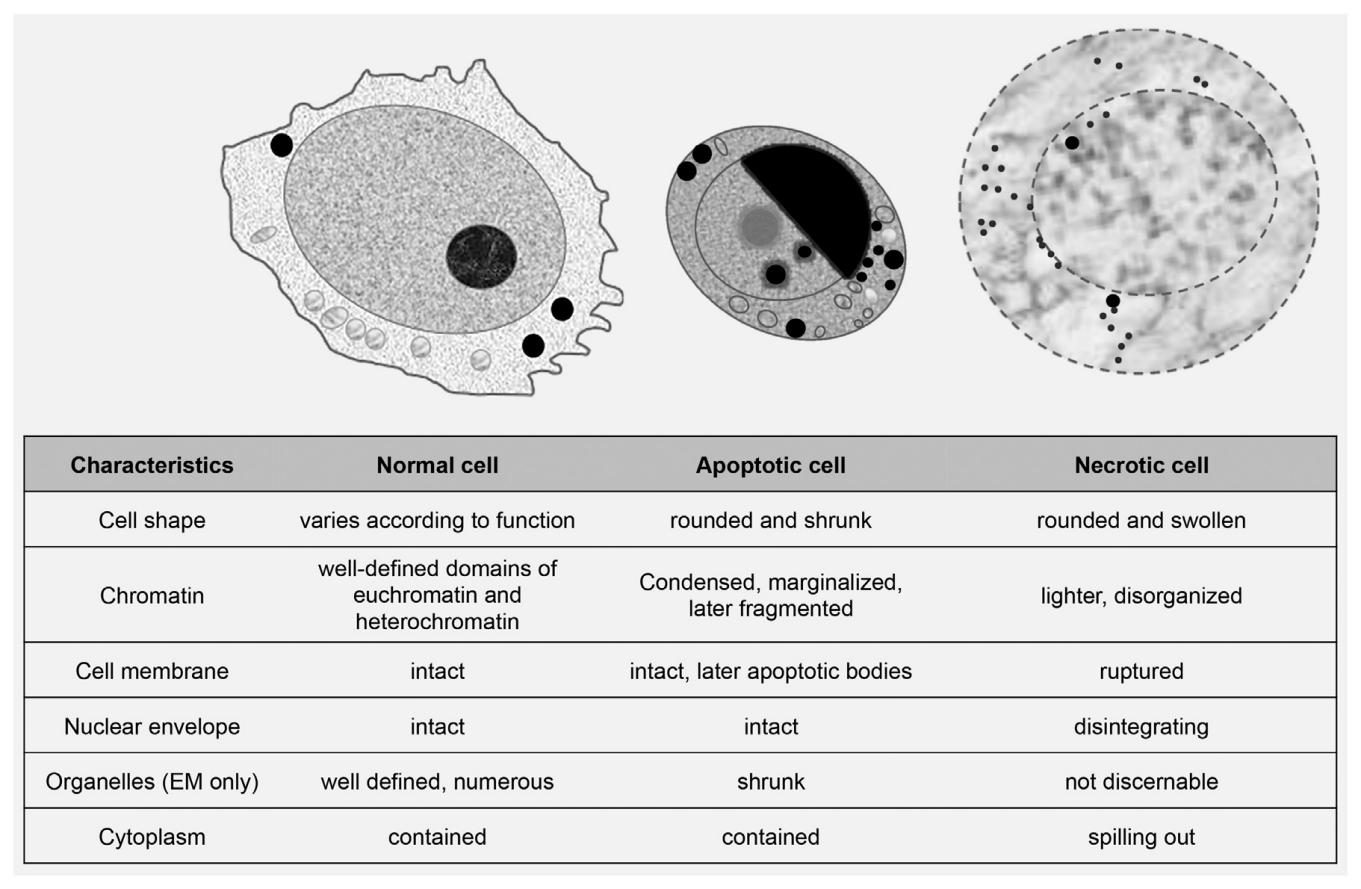
The morphological distinction of apoptosis and necrosis. Schematic drawing and major characteristics. EM, electron microscopy.

The observation that cells can undergo cell death during development was first made in the 1920s (reviewed in 1). The morphology of these dying cells was found to be distinctly different than that observed in necrotic cells (for example, cells subjected to injury). Isolated cells or at most small clusters of cells undergo cell death during normal development. Cells undergoing cell death during embryonic development shrink rather than rupture as seen in necrosis ( Figure 1); cell nuclei condense in what is termed pyknosis (from the Greek *pykno*, meaning thick, compact, or dense) and ultimately fragment. The components of such dying cells are packaged into cytoplasmic membrane–enclosed vesicles awaiting phagocytosis by other cells. In contrast, during necrosis, which can occur during injury and inflammation, larger areas of tissue are usually affected, cells swell, the plasma membrane and nuclear envelope rupture, and cell contents spill out.

The predictable time course and location of the dying cells during development suggested that it was a regulated process and the term programmed cell death was used to indicate this concept. Based on the time course and location, the essential roles of programmed cell death during development—including requirement for tissue invagination and closure, union of body halves, lumen formation, bifurcation, regression of rudimentary organs, and cell differentiation—were inferred ^1^. Later, the electron microscopic characteristics of the dying cells were described and the term apoptosis (from the Greek *apo* + *ptosis*, meaning from + falling) was coined (reviewed in 2). The cell membrane–bound fragments of apoptotic cells were termed apoptotic bodies. Apoptosis is thought to drive morphogenesis by regulating cell number, tissue sculpturing, and deleting structures, including the conversion of solid structures into tubes and vesicles ^3^.

## Regulation of apoptosis

**Figure 2.**
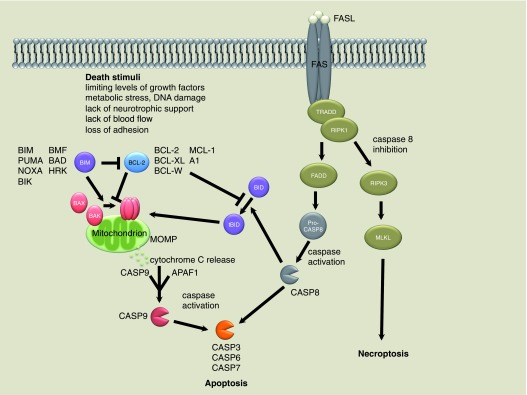
Simplified schematic drawing of the mitochondrial and death receptor apoptotic pathways. MOMP, mitochondrial outer membrane permeabilization.

In the following decades, a large number of researchers worked to decipher the molecular mechanisms that initiate and execute apoptosis during development; they also determined the role of apoptosis in disease conditions (reviewed in 4, 5). Two distinct but ultimately converging pathways initiate apoptosis ^6^: the mitochondrial, intrinsic, or B-cell lymphoma 2 (BCL-2)–regulated pathway and the extrinsic or death receptor pathway ( Figure 2).

The mitochondrial pathway is initiated during development by limiting levels of growth factors, metabolic stress, lack of neurotrophic support, lack of blood flow, or loss of substrate adhesion ( *anoikis*, from the Greek *an* + *oikos*, meaning without + home) and in disease or experimental settings by DNA damage and diverse cytotoxic agents. The response to these stimuli is regulated by BCL-2 protein family members ^4^. In response to death-inducing stimuli, pro-apoptotic members of the family (BIM, PUMA, BID, BMF, NOXA, BIK, BAD, and HRK; collectively termed BH3 [BCL-2 homology domain 3]-only proteins) inhibit the anti-apoptotic BCL-2 family members (BCL-2, MCL-1, BCL-XL, BCL-W, and A1/BFL-1), which under steady-state conditions keep the pro-apoptotic effectors, the multi-BH domain proteins BAX and BAK, in an inactive state. In addition, some BH3-only proteins (for example, BIM and PUMA) have been reported to activate BAX and BAK directly ^4, 5^. Once activated, BAX and BAK form pores within the mitochondrial outer membrane, which leads to mitochondrial outer membrane permeabilization (MOMP) and release of cytochrome C and other apoptogenic factors from the mitochondria into the cytoplasm ( Figure 2).

Cytochrome C associates with APAF-1 and caspase-9 to form the apoptosome and this stimulates caspase-9 activation, which in turn cleaves and thereby activates effector caspase-3, -6, and -7. These effector caspases cleave hundreds of proteins and in certain cases proteolytically activate or inhibit other enzymes. This includes the activation of endonucleases that drive DNA fragmentation and the inhibition of flippases, which causes exposure of “eat me signals” on the plasma membrane. These processes lead to chromatin condensation and packaging of the cell content into apoptotic bodies and removal by phagocytosis.

In the death receptor pathway, apoptosis is initiated by the binding of certain members of the tumor necrosis factor (TNF) family to their cognate receptors that belong to the TNF receptor (TNFR) family (in particular, binding of FAS ligand [FASL] to its receptor FAS). This can set in train two different series of events: if available, pro-caspase-8, which directly activates effector caspase-3, -6 and -7, is activated. The pro-apoptotic BH3-only protein BID, after proteolytic conversion to tBID, can also activate the BAX/BAK-dependent mitochondrial apoptotic pathway to thereby amplify the apoptotic cascade ( Figure 2). If caspase-8 is absent or inhibited (for example, by viral inhibitors of this protease, such as vFLIP), another form of regulated cell death, termed necroptosis ^7^, can be initiated via receptor-interacting serine/threonine-protein kinase 1 (RIPK1), RIPK3, and mixed lineage kinase domain-like pseudokinase (MLKL) ( Figure 2). These alternative fates after the activation of FAS or certain other surface receptors (for example, TNFR1) result in either apoptotic or necroptotic cell death with discernible histological morphologies ( Figure 1). The form of cell death reported to occur during embryonic development resembles apoptotic cell death. Based on histological studies, necroptotic cell death, if it occurred during development, would not be common.

In the following section, we summarize originally proposed roles of apoptotic cell death during development and more recent detailed functional studies that have identified the much more restricted gambit of processes that critically depend on developmental apoptosis. In the conclusion, we propose why developmental apoptosis occurs in many tissues, seemingly without being essential.

## Apoptosis during embryonic development

Histological analyses of developing embryos discovered that pyknotic (dying/dead) cells were present in certain locations at given stages of development. Diverse animal models, from *Caenorhabditis elegans* to *Mus musculus*, were used *.* In *C. elegans*, specific individual cells destined to undergo cell death were identified ^3, 8– 10^. In contrast, clusters of cells undergoing cell death in mouse embryos were noted at specific stages in specific regions, but death did not appear to be pre-programmed to target specific individual cells ( Table 1). In mice, pyknotic cells were detected in a range of tissues, and it was inferred that their death was required for a range of developmental processes, including cavitation, vesicle and tube formation (lens, neural tube, and intestine), fusion of epithelial sheets (neural tube formation, midline body wall, and palate), removal of vestigial tissue (pro-nephros, parts of meso- and meta-nephros, notochord, and Müllerian duct in males and Wolffian duct in females), and tissue cellular homeostasis (summarized in 1).

**Table 1.** Examples of developmental processes proposed to rely on programmed cell death.

Year	Proposed roles of apoptosis in development	Reference
1951	Table of incidences of programmed cell death during development Including vesicle formation (optic vesicle, lens) tube formation by invagination and detachment from epithelial sheets (neural tube, intestine) lumen formation (salivary gland, duodenum, colon, vagina) fusion of epithelial sheets (midline body wall, palate) removal of vestigial tissue (pro-nephros, parts of meso and meta- nephros, notochord, and Müllerian duct in males Wolffian duct in females)	Glücksmann ^1^
1972	Morphological characteristics of developmentally programmed cell death	Kerr *et al.* ^2^
1980	Shaping of the epiblast	Poelmann ^12^
1989	Elimination of redundant cells from the inner cell mass of the blastocyst	Pierce *et al.* ^11^
1992	Kidney development	Koseki *et al.* ^19^
1993	Kidney developing	Coles *et al.* ^20^
1993	Elimination of neural crest from odd-numbered rhombomeres	Graham *et al.* ^13^
1994	Lens vesicle development	Morgenbesser *et al.* ^14^
1995	Lens vesicle development	Pan and Griep ^21^
1995	Pro-amniotic cavity formation	Coucouvanis and Martin ^15^
1996	Digit separation by removing interdigital tissue	Jacobsen *et al.* ^16^
1997	Inner ear morphogenesis	Fekete *et al.* ^22^
1997	Shaping the limbs	Macias *et al.* ^17^
1997	Chondrocytes undergo hypertrophy and apoptosis during enchondral bone development	Amling *et al.* ^23^
1997	Neural tube closure	Weil *et al.* ^18^
2000	Palatal shelf fusion	Martínez-Alvarez *et al.* ^24^
2015	Epithelial folding	Monier *et al.* ^25^

Subsequent reports proposed that apoptotic cell death was essential for normal development in regions where pyknotic cells were observed ( Table 1), including for the elimination of redundant cells from the inner cell mass of the embryonic day 3 (E3.5) blastocyst ^11^, and during the development of the epiblasts at E6 to E7 ^12^, neural crest cells ^13^, lens vesicle ^14^, and the pro-amniotic cavity ^15^, for digit separation by removing interdigital tissue at E13.5 ^16^, shaping the limbs ^17^, neural tube closure ^18^, and elimination of supernumerary neurons during early postnatal development of the brain ^26^.

## Evidence that the mitochondrial apoptotic pathway is essential for mammalian development

Mice lacking individual apoptotic regulators provided evidence for the requirements for specific regulators and suggested that developmental apoptosis was essential for mammalian development ( Table 2).

**Table 2.** Examples of developmental anomalies observed in mice after deletion of genes encoding regulators of apoptosis.

Gene deletion	Major developmental phenotype (major adult phenotype)	Reference
*A1a*	Developmentally normal (accelerated neutrophil apoptosis)	Hamasaki *et al.*, 1998 ^52^
*Apaf1*	Neural tube closure defect, delayed removal of interdigital webs	Cecconi *et al.*, 1998 ^42^ Yoshida *et al.*, 1998 ^43^
*Bak*	Developmentally normal (platelet accumulation)	Lindsten *et al.* 2000 ^53^ Mason *et al.* 2007 ^54^
*Bax*	Developmentally normal (lymphocyte accumulation, male infertility)	Knudson *et al.*, 1995 ^55^
*Bax;Bak* DKO	Defective removal of interdigital webs, vaginal septa, cell homeostasis in the forebrain neurogenic region and in the hematopoietic system	Lindsten *et al.*, 2000 ^53^
*Bax;Bim* DKO	Webbed feet, male infertility (lymphocyte accumulation)	Hutcheson *et al.*, 2005 ^56^
*Bak;Bim* DKO	Developmentally normal (lymphocyte accumulation)	Hutcheson *et al.*, 2005 ^56^
*Bak;Bok* DKO	Developmentally normal	Ke *et al.*, 2013 ^57^
*Bax;Bok* DKO	Developmentally normal (in addition to *Bax* SKO phenotype, number of oocytes increased)	Ke *et al.*, 2013 ^57^
*Bax;Bak;Bok* TKO	Midline fusion defects (exencephaly, spina bifida, omphalocele, cleft face/palate/ lip), webbed feet, vaginal septa, aortic arch defects, renal pelvis encroached by excess tissue, (supernumerary neurons and hematopoietic cells)	Ke *et al.*, 2018 ^35^ Carpio *et al.*, 2016 ^58^
*Bad*	Developmentally normal	Ranger *et al.*, 2003 ^59^ Kelly *et al.*, 2010 ^60^
*Bclx*	Lethality at about E13.5, increased apoptosis of hematopoietic and neuronal cells (decreased platelet half-life)	Motoyama *et al.*, 1995 ^61^ Mason *et al.* 2007 ^54^
*Bclx;Bim* DKO	Loss of BIM rescues hematopoietic and germ cells but not neuronal cell apoptosis in *Bclx ^–/–^* mice	Akhtar *et al.*, 2008 ^62^
*Bcl2*	Developmentally normal (polycystic kidney, loss of lymphocytes)	Veis *et al.*, 1993 ^63^
*Bcl2;Bim* DKO	Developmentally normal (all defects caused by the loss of *Bcl2* are rescued by the concomitant loss of *Bim*, including kidney, lymphocyte, and melanocytes) **	Bouillet *et al.*, 2001 ^64^
*Bik*	Developmentally normal	Coultas *et al.*, 2004 ^65^
*Bim*	About 50% of *Bim ^–/–^* mice are lost during embryogenesis prior to E10; others are developmentally normal and fertile (hematopoietic cell accumulation, autoimmune kidney disease).	Bouillet *et al.*, 1999 ^66^
*Bim;Bmf* DKO	In addition to the *Bim ^–/–^* phenotype, webbed feet, vaginal aplasia	Labi *et al.*, 2014 ^67^
*Bim;Bik* DKO	In addition to the *Bim ^–/–^* phenotype, DKO display (male sterility)	Coultas *et al.*, 2005 ^68^
*Bim;Bad* DKO	In addition to the *Bim ^–/–^* phenotype, slightly more lymphocyte accumulation	Kelly *et al.*, 2010 ^60^
*Bmf*	Vaginal atresia (lymphoid cell accumulation)	Hubner *et al.*, 2010 ^69^ Labi *et al.*, 2008 ^70^,2014 ^67^
*Bok*	Developmentally normal	Ke *et al.*, 2012 ^71^
*Casp3*	Reduced neuronal apoptosis, neural hyperplasias, pre- and postnatal lethality	Kuida *et al.*, 1996 ^72^
Casp3;Casp7 DKO	Neural tube closure defect, heart anomalies	Lakhani *et al.*, 2006 ^46^
*Casp9*	Neural tube closure defect	Hakem *et al.*, 1998 ^45^ Kuida *et al.*, 1998 ^44^
*Hrk*	Developmentally normal	Coultas *et al.*, 2007 ^73^
*Mcl1*	Embryonic lethality at the blastocyst stage prior to implantation	Rinkenberger *et al.*, 2000 ^74^
*Mcl1;Bclx* compound hets	Craniofacial anomalies, including cases of holoprosencephaly, which are rescued by loss of one copy of Bim	Grabow *et al.*, 2018 ^36^
*Mcl1;Bclx* DKO	Nervous system–specific DKO, neuronal apoptosis	Fogarty *et al.*, 2019 ^75^
*Puma*	Developmentally normal	Villunger *et al.*, 2003 ^76^
*Noxa*	Developmentally normal	Villunger *et al.*, 2003 ^76^
*Puma;Noxa* DKO	Developmentally normal	Michalak *et al.*, 2008 ^77^

DKO, double knockout; E, embryonic day; hets, heterozygotes; SKO, single knockout; TKO, triple knockout.

Indeed, from the original large number of developmental processes proposed to require apoptosis to proceed normally ( Table 1), knockout mouse studies in recent years have singled out those developmental processes that do and those that do not require apoptosis ( Table 2). In particular, it has become clear that reducing apoptosis typically causes webbed digits, vaginal septa, and often lymphadenopathy; it commonly causes exencephaly and cleft face or palate and occasionally omphalocele. Therefore, it can be said that the removal of the interdigital webbing, vaginal septa, blood cell homeostasis, neural tube, palate, and body wall closure depends critically on developmental apoptosis. However, whether development leading up to these events or the processes themselves require apoptosis was not discriminated by these studies. Indeed, the sequence of events can be complex. For example, developing neurons can undergo apoptosis before or after target innervation ^27, 28^. However, it appears that the lack or restriction of neurotrophic support may be the cause of neuronal apoptosis on both occasions ^28, 29^.

## Technical difficulties in enumerating apoptotic cells during development

As a corollary of identifying developmental processes that require apoptosis, those processes that occur normally without apoptosis might also have been defined. However, to make such a claim with certainty, the possibility of residual apoptosis occurring would have to be excluded. The difficulties in assessing absolute numbers of apoptotic cells during development can be illustrated by using the example of embryos lacking the tumor suppressor p53, which in response to a number of stress stimuli can induce either cell cycle arrest or apoptosis ^30, 31^. Male mice lacking a functional *Trp53* gene are born in the expected numbers, but female *Trp53 ^–/–^* mice are under-represented because of a partially penetrant failure of neural tube closure ^32, 33^. Originally, electron microscopy ^32^ and terminal deoxynucleotidyl transferase dUTP nick end labeling of DNA fragments (TUNEL) (a technique used to identify cells undergoing apoptosis) on sections ^33^ were employed to determine whether loss of p53 affected developmental apoptosis, but neither method detected a difference between wild-type and *Trp53 ^–/–^* mouse embryos. It was hypothesized that the methods employed may not have been sensitive enough to detect differences ^33^. More recently, developmental apoptosis in *Trp53 ^–/–^* embryos was assessed by TUNEL flow cytometry on single cell suspensions and this method was sufficiently sensitive to detect a difference between *Trp53 ^–/–^* and wild-type ** embryos ^34^. Flow cytometry proved to be a sensitive and precise method that also easily detected differences between wild-type and *Bax ^–/–^;Bak ^–/–^* double knockout (DKO) embryos (total absence of apoptosis) ^35^ or *Mcl1 ^+/–^;Bclx ^+/–^* double heterozygous mice (abnormally increased apoptosis) ^36^. However, the low percentage of apoptotic cells in developing wild-type embryos (only about 1.5% ^34– 36^) does not provide a large dynamic range for detecting a gradual reduction in apoptosis. Indeed, although the more severe developmental abnormalities in mice with combined loss of pro-apoptotic BAX, BAK, and BOK compared with loss of only BAX and BAK suggested that the *Bax ^–/–^;Bak ^–/–^;Bok ^–/–^* triple knockout mice (TKO) should have a greater reduction in developmental apoptosis than *Bax ^–/–^;Bak ^–/–^* DKO mice, flow cytometric analysis was unable to detect significant differences between these two genotypes ^35^. Therefore, a claim of zero apoptosis is hard to support with the current methods. A more sensitive approach may be the use of mice carrying the *CAG-sA5-YFP* transgene, which encodes a fluorescently labeled, secreted form of annexin V. The fluorescently labeled annexin V accumulates to detectable levels when binding to phosphatidylserine exposed on the surface of apoptotic cells ^37^. A caveat here may be that necroptotic cells also stain positive for annexin V ^38^.

## Developmental processes less likely to require apoptosis

It was surprising that apoptosis, given its stereotypic occurrence, was not essential for *C. elegans* development ^8^ and from time to time it was speculated that apoptosis in mammalian development, too, might be less critical than its prevalence might suggest ^39^. Throughout the decades of intense research on the role of apoptosis in a number of developmental processes, information surfaced indicating that, despite previous inferences, apoptosis was not required for specific processes. An example is the role of apoptosis in the formation of the pro-amniotic cavity, which was viewed as a model of cavity formation/tube formation by apoptosis and supported by work in *in vivo* and culture models ^15, 40, 41^ but was later called into question by the development of embryos past this point in the absence of detectable apoptosis ^35^. Similarly, apoptosis had been observed in the ridge of the closing neural folds and therefore it had been proposed that neural tube closure required apoptotic cell death at the point where the two neural folds met. This was supported by the observation that the application of caspase inhibitors at a critical time point blocked neural tube closure ^18^. On the surface, this conclusion appears to be amply supported by the many knockout mouse mutants of apoptotic regulators that display exencephaly or spina bifida or both ( *Bax;Bak;Bok* TKO ^35^, *Trp53* KO ^32– 34^, *Apaf1* KO ^42, 43^, *Casp9* KO ^44, 45^, and *Casp3;Casp7* DKO ^46^). However, one study presented evidence that apoptosis is not actually required for the specific time of neural tube closure and that lack of apoptosis did not affect tissue remodeling and separation of the neural tube from the surface ectoderm at the fusion site ^47^. Similarly, it is not clear whether apoptosis is required at the specific time of palate fusion ^35, 48– 51^.

The studies showing that neural tube closure and palate fusion can occur without developmental apoptosis raise the question of why so many mouse mutants of critical constituents of the mitochondrial apoptotic pathway display exencephaly, spina bifida, and cleft palate ( Table 2). A possible explanation is that it is not the process of tissue fusion that requires apoptosis but rather that apoptosis is important to arrive at body halves that are of a size compatible with the fusion process.

## Death receptor pathway–induced apoptosis and necroptosis

Activation of the death receptor pathway can lead to apoptosis via caspase-8 activation or, when caspase-8 is inhibited, to necroptosis via RIPK1/RIPK3/MLKL activation ( Figure 2). Death receptor pathway–mediated apoptosis is morphologically similar to mitochondrial apoptosis, but necroptosis is distinct, resembling necrosis. Disturbance of the normal regulation of death receptor–induced cell death pathways can result in developmental lethality. Mouse embryos lacking caspase-8 display prenatal lethality ^78^ and mice lacking RIPK1 die shortly after birth ^79^ whereas RIPK3-deficient mice develop normally ^80^. However, if both death receptor–activated processes (apoptosis and necroptosis) are inactivated by simultaneously deleting genes necessary for each process, the resulting mice are viable ** (for example, *Casp8*; *Ripk3* DKO ^81, 82^; *Casp8*; *Mlkl* or *Casp8*; *Fadd* DKO ^83^; and *Ripk1;Ripk3*; *Casp8* or *Ripk1;Ripk3*; *FADD* TKO ^84^). The studies using these compound mutant mice provided evidence that death receptor–mediated apoptosis is not required for normal mouse development; rather, a balance between caspase-8 and RIPK1/RIPK3 activity needs to be maintained to prevent MLKL activation and consequent necroptosis. The observation that mice lacking MLKL ^85^, the essential mediator of necroptosis, develop normally reveals that this programmed cell death process is not required for embryogenesis. In agreement with these findings, early histological studies did not detect substantial involvement of necrotic processes in mammalian development.

## Other forms of cell death and cell removal mechanisms

The most abundant form of developmental cell death appears to be apoptosis, and more specifically mitochondrial apoptosis, as the death receptor pathway does not appear to be essential for normal development (discussed above). Furthermore, reduction of mitochondrial apoptosis to below current detection levels by simultaneous deletion of *Bax*, *Bak*, and *Bok* did not appear to cause a compensatory increase in necroptosis, pyroptosis, or autophagy ^35^, suggesting that these other forms of cell death may not be poised to compensate for a deficiency of the mitochondrial apoptotic pathway.

A number of alternative cell removal processes have been proposed to play a role during development, including entosis ^86^, autophagy ^87, 88^, cell extrusion from epithelia ^1, 89, 90^, and cell senescence ^91, 92^. However, their prominence and roles during mammalian development remain to be determined. Autophagy is morphologically distinct from apoptosis ^93^ and so could be assessed and its frequency could be compared to apoptosis. An overlap between apoptosis and entosis exists in so far as apoptotic cells are eventually engulfed by other cells. Whether mammalian cells are extruded from epithelia (for example, in the intestine) before or after they have initiated cell death appears contentious ^1, 94, 95^, and the ultimate fate of senescent cells seems to be cell death, possibly mostly through apoptosis ^92^.

Overall, the mitochondrial apoptotic pathway appears to be the major form of developmental cell death. However, it should be noted that low prevalence of a given process does not necessarily imply little importance. Accordingly, it is possible that in specific cases these alternative forms of cell death play an essential role during mammalian development, which remains to be functionally tested. This will probably require the generation and analysis of mice deficient in multiple cell death pathways and possibly even mice lacking several cell death pathways plus other processes (for example, cell senescence) to uncover redundancy.

## Open questions

The following open questions exist in the field of developmental apoptosis.

(1) The field might benefit from the results of a comprehensive, sensitive, and quantitative analysis of apoptosis throughout the sequence of mammalian development that either does not rely on tissue sections or uses sophisticated methods of three-dimensional reconstruction and numerical assessment.
(2) Once a sensitive technical approach to (1) has been developed, the question of whether developmental apoptosis is dynamically regulated during development in response to disruptions (for example, of proliferation or cell survival) could be answered.
(3) If similarly sensitive and quantitative detection methods were developed for other forms of cell death, their relative importance compared with apoptosis could be assessed.
(4) The role of a number of BCL-2 family members in development remains to be determined, while single knockout of the genes encoding these does not result in overt phenotypic anomalies, which is possibly due to functional redundancies.
(5) In addition, based on the theory of evolution, one would expect that even these BCL-2 family members have essential functions (that provide a selective advantage), which remain to be discovered.

## Conclusions

The available data identify developmental processes that are clearly dependent on functional apoptosis, whereas other processes previously thought to depend on programmed cell death do not appear to be as reliant on apoptosis.

Processes apparently independent of apoptosis based on normal embryonic and fetal development in the combined absence of BAX, BAK, and BOK include, for example, the formation of the pro-amniotic cavity, epiblast, intestine, and lens development as well as enchondral ossification. (For more details, see Table S2 in Ke *et al*., 2018 ^35^.) However, the caveat that residual apoptosis not detectable by current methods (discussed in the preceding sections) needs to be kept in mind.

It is certainly curious that a complex and regulated process, such as apoptosis, occurs in an evolutionary conserved manner during development, including in places where it appears to be largely or completely dispensable. One possibility is that apoptosis occurs as a by-product of other processes; for example, in the process of tissue remodeling, cells might lose their contact to the extracellular matrix and, without this survival signal, might die by apoptosis (anoikis).

Another possible explanation is that a twofold control of developing tissue size and cell population homeostasis by maintaining a balance between proliferation and cell death affords not just one but two cellular mechanisms of fine-tuning cell number. This would provide a more robust regulation, less prone to errors and variation in response to negative impact.

Indeed, mammalian and other embryos appear to have an astounding capacity to compensate for the loss of cells, certainly by upregulating cell proliferation ^96, 97^ but perhaps also by actively downregulating developmental apoptosis. It can be assumed, on the basis of its evolutionary conservation, that apoptotic cell death, even in regions where it is not absolutely required, provides a competitive advantage.

## References

[1] Glucksmann A: Cell deaths in normal vertebrate ontogeny. *Biol Rev Camb Philos Soc.* 1951;26(1):59–86. 10.1111/j.1469-185x.1951.tb00774.x 24540363

[2] Kerr JFR Wyllie AH Currie AR: Apoptosis: A Basic Biological Phenomenon with Wide-ranging Implications in Tissue Kinetics. *Br J Cancer.* 1972;26(4):239–57. 10.1038/bjc.1972.33 4561027 PMC2008650

[3] Fuchs Y Steller H: Programmed cell death in animal development and disease. *Cell.* 2011;147(4):742–58. 10.1016/j.cell.2011.10.033 22078876 PMC4511103

[4] Czabotar PE Lessene G Strasser A: Control of apoptosis by the BCL-2 protein family: Implications for physiology and therapy. *Nat Rev Mol Cell Biol.* 2014;15(1):49–63. 10.1038/nrm3722 24355989

[5] Green DR Kroemer G: The pathophysiology of mitochondrial cell death. *Science.* 2004;305(5684):626–9. 10.1126/science.1099320 15286356

[6] Strasser A Harris AW Huang DC: Bcl-2 and Fas/APO-1 regulate distinct pathways to lymphocyte apoptosis. *EMBO J.* 1995;14(24):6136–47. 10.1002/j.1460-2075.1995.tb00304.x 8557033 PMC394738

[7] Shan B Pan H Najafov A: Necroptosis in development and diseases. *Genes Dev.* 2018;32(5-6):327–40. 10.1101/gad.312561.118 29593066 PMC5900707

[8] Ellis H Horvitz HR: Genetic control of programmed cell death in the nematode C. elegans. *Cell.* 1986;44(6):817–29. 10.1016/0092-8674(86)90004-8 3955651

[9] Ellis RE Yuan JY Horvitz HR: Mechanisms and functions of cell death. *Annu Rev Cell Biol.* 1991;7:663–98. 10.1146/annurev.cb.07.110191.003311 1809356

[10] Strasser A Vaux DL: Viewing BCL2 and cell death control from an evolutionary perspective. *Cell Death Differ.* 2018;25(1):13–20. 10.1038/cdd.2017.145 29099481 PMC5729521

[11] Pierce GB Lewellyn AL Parchment RE: Mechanism of programmed cell death in the blastocyst. *Proc Natl Acad Sci U S A.* 1989;86(10):3654–8. 10.1073/pnas.86.10.3654 2726743 PMC287196

[12] Poelmann RE: Differential mitosis and degeneration patterns in relation to the alterations in the shape of the embryonic ectoderm of early post-implantation mouse embryos. *J Embryol Exp Morphol.* 1980;55:33–51. 7373201

[13] Graham A Heyman I Lumsden A: Even-numbered rhombomeres control the apoptotic elimination of neural crest cells from odd-numbered rhombomeres in the chick hindbrain. *Development.* 1993;119(1):233–45. 8275859 10.1242/dev.119.1.233

[14] Morgenbesser SD Williams BO Jacks T: p53-dependent apoptosis produced by Rb-deficiency in the developing mouse lens. *Nature.* 1994;371(6492):72–4. 10.1038/371072a0 8072529

[15] Coucouvanis E Martin GR: Signals for death and survival: A two-step mechanism for cavitation in the vertebrate embryo. *Cell.* 1995;83(2):279–87. 10.1016/0092-8674(95)90169-8 7585945

[16] Jacobsen MD Weil M Raff MC: Role of Ced-3/ICE-family proteases in staurosporine-induced programmed cell death. *J Cell Biol.* 1996;133(5):1041–51. 10.1083/jcb.133.5.1041 8655577 PMC2120856

[17] Macias D Gañan Y Sampath TK: Role of BMP-2 and OP-1 (BMP-7) in programmed cell death and skeletogenesis during chick limb development. *Development.* 1997;124(6):1109–17. 9102298 10.1242/dev.124.6.1109

[18] Weil M Jacobson MD Raff MC: Is programmed cell death required for neural tube closure? *Current Biology.* 1997;7(4):281–4. 10.1016/s0960-9822(06)00125-4 9094312

[19] Koseki C Herzlinger D al-Awqati Q: Apoptosis in metanephric development. *J Cell Biol.* 1992;119(5):1327–33. 10.1083/jcb.119.5.1327 1447305 PMC2289732

[20] Coles HS Burne JF Raff MC: Large-scale normal cell death in the developing rat kidney and its reduction by epidermal growth factor. *Development.* 1993;118(3):777–84. 8076517 10.1242/dev.118.3.777

[21] Pan H Griep AE: Temporally distinct patterns of p53-dependent and p53-independent apoptosis during mouse lens development. *Genes Dev.* 1995;9(17):2157–69. 10.1101/gad.9.17.2157 7657167

[22] Fekete DM Homburger SA Waring MT: Involvement of programmed cell death in morphogenesis of the vertebrate inner ear. *Development.* 1997;124(12):2451–61. 9199371 10.1242/dev.124.12.2451

[23] Amling M Neff L Tanaka S: Bcl-2 Lies Downstream of Parathyroid Hormone-related Peptide in a Signaling Pathway That Regulates Chondrocyte Maturation during Skeletal Development. *J Cell Biol.* 1997;136(1):205–13. 10.1083/jcb.136.1.205 9008714 PMC2132464

[24] Martínez-Alvarez C Tudela C Pérez-Miguelsanz J: Medial edge epithelial cell fate during palatal fusion. *Dev Biol.* 2000;220(2):343–57. 10.1006/dbio.2000.9644 10753521

[25] Monier B Gettings M Gay G: Apico-basal forces exerted by apoptotic cells drive epithelium folding. *Nature.* 2015;518(7538):245–8. 10.1038/nature14152 25607361

[26] White LD Barone S: Qualitative and quantitative estimates of apoptosis from birth to senescence in the rat brain. *Cell Death Differ.* 2001;8(4):345–56. 10.1038/sj.cdd.4400816 11550086

[27] Sun W Gould TW Vinsant S: Neuromuscular Development after the Prevention of Naturally Occurring Neuronal Death by Bax Deletion. *J Neurosci.* 2003;23(19):7298–310. 10.1523/JNEUROSCI.23-19-07298.2003 12917363 PMC6740454

[28] Fariñas I Yoshida CK Backus C: Lack of Neurotrophin-3 Results in Death of Spinal Sensory Neurons and Premature Differentiation of Their Precursors. *Neuron.* 1996;17(6):1065–78. 10.1016/s0896-6273(00)80240-8 8982156 PMC2758230

[29] Deppmann CD Mihalas S Sharma N: A model for neuronal competition during development. *Science.* 2008;320(5874):369–73. 10.1126/science.1152677 18323418 PMC3612357

[30] Vousden KH Lane DP: p53 in health and disease. *Nat Rev Mol Cell Biol.* 2007;8(4):275–83. 10.1038/nrm2147 17380161

[31] Kastenhuber ER Lowe SW: Putting p53 in Context. *Cell.* 2017;170(6):1062–78. 10.1016/j.cell.2017.08.028 28886379 PMC5743327

[32] Armstrong JF Kaufman MH Harrison DJ: High-frequency developmental abnormalities in p53-deficient mice. *Current Biology.* 1995;5(8):931–6. 10.1016/s0960-9822(95)00183-7 7583151

[33] Sah VP Attardi LD Mulligan GJ: A subset of p53-deficient embryos exhibit exencephaly. *Nat Genet.* 1995;10(2):175–80. 10.1038/ng0695-175 7663512

[34] Delbridge ARD Kueh AJ Ke F: Loss of p53 Causes Stochastic Aberrant X-Chromosome Inactivation and Female-Specific Neural Tube Defects. *Cell Rep.* 2019;27(2):442–454.e5. 10.1016/j.celrep.2019.03.048 30970248

[35] Ke FFS Vanyai HK Cowan AD: Embryogenesis and Adult Life in the Absence of Intrinsic Apoptosis Effectors BAX BAK, and BOK. *Cell.* 2018;173(5):1217–1230.e17. 10.1016/j.cell.2018.04.036 29775594

[36] Grabow S Kueh AJ Ke F: Subtle Changes in the Levels of BCL-2 Proteins Cause Severe Craniofacial Abnormalities. *Cell Rep.* 2018;24(12):3285–3295.e4. 10.1016/j.celrep.2018.08.048 30232009

[37] Martínez-Lagunas K Yamaguchi Y Becker C: In vivo detection of programmed cell death during mouse heart development. *Cell Death Differ.* 2019;88:347. 10.1038/s41418-019-0426-2 31570857 PMC7205869

[38] Gong YN Guy C Olauson H: ESCRT-III Acts Downstream of MLKL to Regulate Necroptotic Cell Death and Its Consequences. *Cell.* 2017;169(2):286–300.e16. 10.1016/j.cell.2017.03.020 28388412 PMC5443414

[39] Tuzlak S Kaufmann T Villunger A: Interrogating the relevance of mitochondrial apoptosis for vertebrate development and postnatal tissue homeostasis. *Genes Dev.* 2016;30(19):2133–51. 10.1101/gad.289298.116 27798841 PMC5088563

[40] Brown D Yu BD Joza N: Loss of *Aif* function causes cell death in the mouse embryo, but the temporal progression of patterning is normal. *Proc Natl Acad Sci U S A.* 2006;103(26):9918–23. 10.1073/pnas.0603950103 16788063 PMC1502554

[41] Joza N Susin SA Daugas E: Essential role of the mitochondrial apoptosis-inducing factor in programmed cell death. *Nature.* 2001;410(6828):549–54. 10.1038/35069004 11279485

[42] Cecconi F Alvarez-Bolado G Meyer BI: Apaf1 (CED-4 homolog) regulates programmed cell death in mammalian development. *Cell.* 1998;94(6):727–37. 10.1016/s0092-8674(00)81732-8 9753320

[43] Yoshida H Kong YY Yoshida R: Apaf1 is required for mitochondrial pathways of apoptosis and brain development. *Cell.* 1998;94(6):739–50. 10.1016/s0092-8674(00)81733-x 9753321

[44] Kuida K Haydar TF Kuan CY: Reduced apoptosis and cytochrome c-mediated caspase activation in mice lacking caspase 9. *Cell.* 1998;94(3):325–37. 10.1016/s0092-8674(00)81476-2 9708735

[45] Hakem R Hakem A Duncan GS: Differential requirement for caspase 9 in apoptotic pathways *in vivo*. *Cell.* 1998;94(3):339–52. 10.1016/s0092-8674(00)81477-4 9708736

[46] Lakhani SA Masud A Kuida K: Caspases 3 and 7: key mediators of mitochondrial events of apoptosis. *Science.* 2006;311(5762):847–51. 10.1126/science.1115035 16469926 PMC3738210

[47] Massa V Savery D Ybot-Gonzalez P: Apoptosis is not required for mammalian neural tube closure. *Proc Natl Acad Sci U S A.* 2009;106(20):8233–8. 10.1073/pnas.0900333106 19420217 PMC2688898

[48] Cuervo R Valencia C Chandraratna RA: Programmed cell death is required for palate shelf fusion and is regulated by retinoic acid. *Dev Biol.* 2002;245(1):145–56. 10.1006/dbio.2002.0620 11969262

[49] Fitchett JE Hay ED: Medial edge epithelium transforms to mesenchyme after embryonic palatal shelves fuse. *Dev Biol.* 1989;131(2):455–74. 10.1016/s0012-1606(89)80017-x 2463946

[50] Shuler CF Halpern DE Guo Y: Medial edge epithelium fate traced by cell lineage analysis during epithelial-mesenchymal transformation *in vivo*. *Dev Biol.* 1992;154(2):318–30. 10.1016/0012-1606(92)90071-n 1385235

[51] Takahara S Takigawa T Shiota K: Programmed cell death is not a necessary prerequisite for fusion of the fetal mouse palate. *Int J Dev Biol.* 2004;48(1):39–46. 10.1387/ijdb.15005573 15005573

[52] Hamasaki A Sendo F Nakayama K: Accelerated neutrophil apoptosis in mice lacking A1-a, a subtype of the *bcl-2*-related A1 gene. *J Exp Med.* 1998;188(11):1985–92. 10.1084/jem.188.11.1985 9841913 PMC2212378

[53] Lindsten T Ross AJ King A: The Combined Functions of Proapoptotic Bcl-2 Family Members Bak and Bax Are Essential for Normal Development of Multiple Tissues. *Mol Cell.* 2000;6(6):1389–99. 10.1016/S1097-2765(00)00136-2 11163212 PMC3057227

[54] Mason KD Carpinelli MR Fletcher JI: Programmed anuclear cell death delimits platelet life span. *Cell.* 2007;128(6):1173–86. 10.1016/j.cell.2007.01.037 17382885

[55] Knudson CM Tung KS Tourtellotte WG: Bax-Deficient Mice with Lymphoid Hyperplasia and Male Germ Cell Death. *Science.* 1995;270(5233):96–9. 10.1126/science.270.5233.96 7569956

[56] Hutcheson J Scatizzi JC Bickel E: Combined loss of proapoptotic genes Bak or Bax with Bim synergizes to cause defects in hematopoiesis and in thymocyte apoptosis. *J Exp Med.* 2005;201(12):1949–60. 10.1084/jem.20041484 15967824 PMC2212027

[57] Ke F Bouillet P Kaufmann T: Consequences of the combined loss of BOK and BAK or BOK and BAX. *Cell Death Dis.* 2013;4:e650. 10.1038/cddis.2013.176 23744350 PMC3698543

[58] Carpio MA Michaud M Zhou W: Reply to Fernandez-Marrero *et al.*: Role of BOK at the intersection of endoplasmic reticulum stress and apoptosis regulation. *Proc Natl Acad Sci U S A.* 2016;113(5):E494–E495. 10.1073/pnas.1521979113 26811485 PMC4747777

[59] Ranger AM Zha J Harada H: *Bad*-deficient mice develop diffuse large B cell lymphoma. *Proc Natl Acad Sci U S A.* 2011;100(16):9324–9. 10.1073/pnas.1533446100 12876200 PMC170917

[60] Kelly PN White MJ Goschnick MW: Individual and overlapping roles of BH3-only proteins Bim and Bad in apoptosis of lymphocytes and platelets and in suppression of thymic lymphoma development. *Cell Death Differ.* 2010;17(10):1655–64. 10.1038/cdd.2010.43 20431598 PMC2953537

[61] Motoyama N Wang F Roth K: Massive cell death of immature hematopoietic cells and neurons in Bcl-x-deficient mice. *Science.* 1995;267(5203):1506–10. 10.1126/science.7878471 7878471

[62] Akhtar RS Klocke BJ Strasser A: Loss of BH3-only protein Bim inhibits apoptosis of hemopoietic cells in the fetal liver and male germ cells but not neuronal cells in bcl-x-deficient mice. *J Histochem Cytochem.* 2008;56(10):921–7. 10.1369/jhc.2008.951749 18606610 PMC2544614

[63] Veis DJ Sorenson CM Shutter JR: Bcl-2-deficient mice demonstrate fulminant lymphoid apoptosis, polycystic kidneys, and hypopigmented hair. *Cell.* 1993;75(2):229–40. 10.1016/0092-8674(93)80065-M 8402909

[64] Bouillet P Cory S Zhang LC: Degenerative disorders caused by Bcl-2 deficiency prevented by loss of its BH3-only antagonist Bim. *Dev Cell.* 2001;1(5):645–53. 10.1016/S1534-5807(01)00083-1 11709185

[65] Coultas L Bouillet P Stanley EG: Proapoptotic BH3-only Bcl-2 family member Bik/Blk/Nbk is expressed in hemopoietic and endothelial cells but is redundant for their programmed death. *Mol Cell Biol.* 2004;24(4):1570–81. 10.1128/MCB.24.4.1570-1581.2004 14749373 PMC344198

[66] Bouillet P Metcalf D Huang DC: Proapoptotic Bcl-2 relative Bim required for certain apoptotic responses, leukocyte homeostasis, and to preclude autoimmunity. *Science.* 1999;286(5445):1735–8. 10.1126/science.286.5445.1735 10576740

[67] Labi V Woess C Tuzlak S: Deregulated cell death and lymphocyte homeostasis cause premature lethality in mice lacking the BH3-only proteins Bim and Bmf. *Blood.* 2014;123(17):2652–62. 10.1182/blood-2013-11-537217 24632712 PMC3999752

[68] Coultas L Bouillet P Loveland KL: Concomitant loss of proapoptotic BH3-only Bcl-2 antagonists Bik and Bim arrests spermatogenesis. *EMBO J.* 2005;24(22):3963–73. 10.1038/sj.emboj.7600857 16270031 PMC1283956

[69] Hübner A Cavanagh-Kyros J Rincon M: Functional cooperation of the proapoptotic Bcl2 family proteins Bmf and Bim *in vivo*. *Mol Cell Biol.* 2009;30(1):98–105. 10.1128/MCB.01155-09 19841067 PMC2798311

[70] Labi V Erlacher M Kiessling S: Loss of the BH3-only protein Bmf impairs B cell homeostasis and accelerates gamma irradiation-induced thymic lymphoma development. *J Exp Med.* 2008;205(3):641–55. 10.1084/jem.20071658 18299399 PMC2275386

[71] Ke F Voss A Kerr JB: BCL-2 family member BOK is widely expressed but its loss has only minimal impact in mice. *Cell Death Differ.* 2012;19(6):915–25. 10.1038/cdd.2011.210 22281706 PMC3354060

[72] Kuida K Zheng TS Na S: Decreased apoptosis in the brain and premature lethality in CPP32-deficient mice. *Nature.* 1996;384(6607):368–72. 10.1038/384368a0 8934524

[73] Coultas L Terzano S Thomas T: Hrk/DP5 contributes to the apoptosis of select neuronal populations but is dispensable for haematopoietic cell apoptosis. *J Cell Sci.* 2007;120(Pt 12):2044–52. 10.1242/jcs.002063 17535852 PMC2795636

[74] Rinkenberger JL Horning S Klocke B: Mcl-1 deficiency results in peri-implantation embryonic lethality. *Genes Dev.* 2000;14(1):23–7. 10640272 PMC316347

[75] Fogarty LC Flemmer RT Geizer BA: Mcl-1 and Bcl-xL are essential for survival of the developing nervous system. *Cell Death Differ.* 2019;26(8):1501–15. 10.1038/s41418-018-0225-1 30361616 PMC6748126

[76] Villunger A Michalak EM Coultas L: p53- and drug-induced apoptotic responses mediated by BH3-only proteins puma and noxa. *Science.* 2003;302(5647):1036–8. 10.1126/science.1090072 14500851

[77] Michalak EM Villunger A Adams JM: In several cell types tumour suppressor p53 induces apoptosis largely via Puma but Noxa can contribute. *Cell Death Differ.* 2008;15(6):1019–29. 10.1038/cdd.2008.16 18259198 PMC2974267

[78] Varfolomeev EE Schuchmann M Luria V: Targeted disruption of the mouse Caspase 8 gene ablates cell death induction by the TNF receptors, Fas/Apo1, and DR3 and is lethal prenatally. *Immunity.* 1998;9(2):267–76. 10.1016/s1074-7613(00)80609-3 9729047

[79] Kelliher MA Grimm S Ishida Y: The death domain kinase RIP mediates the TNF-induced NF-kappaB signal. *Immunity.* 1998;8(3):297–303. 10.1016/s1074-7613(00)80535-x 9529147

[80] Newton K Sun X Dixit VM: Kinase RIP3 is dispensable for normal NF-kappa Bs, signaling by the B-cell and T-cell receptors, tumor necrosis factor receptor 1, and Toll-like receptors 2 and 4. *Mol Cell Biol.* 2004;24(4):1464–9. 10.1128/mcb.24.4.1464-1469.2004 14749364 PMC344190

[81] Kaiser WJ Upton JW Long AB: RIP3 mediates the embryonic lethality of caspase-8-deficient mice. *Nature.* 2011;471(7338):368–72. 10.1038/nature09857 21368762 PMC3060292

[82] Oberst A Dillon CP Weinlich R: Catalytic activity of the caspase-8-FLIP(L) complex inhibits RIPK3-dependent necrosis. *Nature.* 2011;471(7338):363–7. 10.1038/nature09852 21368763 PMC3077893

[83] Alvarez-Diaz S Dillon CP Lalaoui N: The Pseudokinase MLKL and the Kinase RIPK3 Have Distinct Roles in Autoimmune Disease Caused by Loss of Death-Receptor-Induced Apoptosis. *Immunity.* 2016;45(3):513–26. 10.1016/j.immuni.2016.07.016 27523270 PMC5040700

[84] Dillon CP Weinlich R Rodriguez DA: RIPK1 blocks early postnatal lethality mediated by caspase-8 and RIPK3. *Cell.* 2014;157(5):1189–202. 10.1016/j.cell.2014.04.018 24813850 PMC4068710

[85] Murphy JM Czabotar PE Hildebrand JM: The pseudokinase MLKL mediates necroptosis via a molecular switch mechanism. *Immunity.* 2013;39(3):443–53. 10.1016/j.immuni.2013.06.018 24012422

[86] Overholtzer M Mailleux AA Mouneimne G: A nonapoptotic cell death process, entosis, that occurs by cell-in-cell invasion. *Cell.* 2007;131(5):966–79. 10.1016/j.cell.2007.10.040 18045538

[87] Fimia GM Stoykova A Romagnoli A: Ambra1 regulates autophagy and development of the nervous system. *Nature.* 2007;447(7148):1121–5. 10.1038/nature05925 17589504

[88] Qu X Zou Z Sun Q: Autophagy gene-dependent clearance of apoptotic cells during embryonic development. *Cell.* 2007;128(5):931–46. 10.1016/j.cell.2006.12.044 17350577

[89] Denning DP Hatch V Horvitz HR: Programmed elimination of cells by caspase-independent cell extrusion in *C. elegans*. *Nature.* 2012;488(7410):226–30. 10.1038/nature11240 22801495 PMC3416925

[90] Eisenhoffer GT Loftus PD Yoshigi M: Crowding induces live cell extrusion to maintain homeostatic cell numbers in epithelia. *Nature.* 2012;484(7395):546–9. 10.1038/nature10999 22504183 PMC4593481

[91] Muñoz-Espín D Cañamero M Maraver A: Programmed cell senescence during mammalian embryonic development. *Cell.* 2013;155(5):1104–18. 10.1016/j.cell.2013.10.019 24238962

[92] Storer M Mas A Robert-Moreno A: Senescence is a developmental mechanism that contributes to embryonic growth and patterning. *Cell.* 2013;155(5):1119–30. 10.1016/j.cell.2013.10.041 24238961

[93] Kroemer G Galluzzi L Vandenabeele P: Classification of cell death: recommendations of the Nomenclature Committee on Cell Death 2009. *Cell Death Differ.* 2009;16(1):3–11. 10.1038/cdd.2008.150 18846107 PMC2744427

[94] Abud HE Heath JK: Detecting apoptosis during the formation of polarized intestinal epithelium in organ culture. *Cell Death Differ.* 2004;11(7):788–9. 10.1038/sj.cdd.4401402 15002039

[95] Michael M Meiring JCM Acharya BR: Coronin 1B Reorganizes the Architecture of F-Actin Networks for Contractility at Steady-State and Apoptotic Adherens Junctions. *Dev Cell.* 2016;37(1):58–71. 10.1016/j.devcel.2016.03.008 27046832

[96] Fogarty CE Bergmann A: Killers creating new life: caspases drive apoptosis-induced proliferation in tissue repair and disease. *Cell Death Differ.* 2017;24(8):1390–400. 10.1038/cdd.2017.47 28362431 PMC5520457

[97] Freret-Hodara B Cui Y Griveau A: Enhanced Abventricular Proliferation Compensates Cell Death in the Embryonic Cerebral Cortex. *Cereb Cortex.* 2017;27(10):4701–18. 10.1093/cercor/bhw264 27620979

